# Genome sequences of four bacterial strains isolated from the phyllosphere of *Mangifera indica* trees in the polluted tropical city of Medellín, Colombia

**DOI:** 10.1128/mra.00952-26

**Published:** 2026-08-24

**Authors:** Luisa María Múnera Porras, Tatiana Muñoz, Nancy J. Pino, Miguel Pérez, João Paulo de Oliveira, Rafaella Cardoso-Sichieri, Amanda Rodrigues, Leonardo Cardoso Alves, Admilton Gonçalves de Oliveira Junior

**Affiliations:** 1 Research Group on Health and Sustainability, School of Microbiology, University of Antioquia 27983, Medellín, Colombia; 2 Diagnostic and Pollution Control Research Group (GDCON), Faculty of Engineering, University of Antioquia 27983, Medellín, Colombia; 3 Biosciences Research Group, Faculty of Health Sciences, University Institution Colegio Mayor de Antioquia 119995, Medellín, Colombia; 4 Laboratory of Microbial Biotechnology (LABIM), Department of Microbiology, CCB, State University of Londrina 37894 , Paraná, Brazil; California State University San Marcos, San Marcos, California, USA

**Keywords:** phyllosphere bacteria, *Mangifera indica*, whole-genome sequencing, atmospheric pollution

## Abstract

Complete and draft genome sequences of four phyllosphere-associated bacterial strains (*Microbacterium radiodurans*, *Brachybacterium rhamnosum*, *Sphingomonas citri*, and *Curtobacterium* sp.) isolated from *Mangifera indica* leaves in polluted Medellín, Colombia, are presented. These resources enable future studies on plant-microbe interactions and phyllosphere microbial mediation of atmospheric pollutants under urban stress.

## ANNOUNCEMENT

Urban air pollution is a major environmental challenge in cities associated with particulate matter and polycyclic aromatic hydrocarbons (PAHs) that affect human health and ecosystems (1–3). It is the second leading cause of mortality from noncommunicable diseases after tobacco use (4). Interest in urban vegetation and its impact on air quality has risen (5, 6), and microorganisms inhabiting the aerial surfaces of plants are viewed as potential contributors to phylloremediation through the microbial mediation of pollutant mitigation (7–10).

Complete genome sequences of two bacterial strains and draft genome sequences of other two bacterial strains isolated from the phyllosphere of *Mangifera indica* sampled in polluted urban Medellín (Antioquia, Colombia) are reported.

Using a perimeter-based sampling strategy, 50 g of aseptically-collected, apparently healthy, petiole-free leaves from the adult *M. indica* tree, located in a high-traffic area (6.2677°N, 75.5688°W), was sampled in November 2024. Leaves were washed (200 rpm, 10 min, ~24°C) in phosphate buffer supplemented with 50 mM EDTA to detach epiphytic microorganisms. Aliquots (100 µL) of the resulting wash suspension were plated on R2A agar and TSA 0.1×, incubated at 30°C, and inspected for 72 h. Distinct morphotypes were selected, whereas taxa reported as pathogenic or antibiotic-resistant were excluded to obtain representative phyllosphere-associated bacteria. Strains T01M1 and T01M4 were recovered from 0.1 × TSA, whereas strains RM6 and RM9 were recovered from R2A agar.

Selected isolates were grown aerobically in liquid TSA (30°C, 48 h), and genomic DNA was extracted using the DNeasy PowerSoil kit (Qiagen, Germany). Whole-genome sequencing was performed using an Oxford Nanopore MinION MK1B, the Rapid Sequencing DNA v14 kit, and FLO-MIN114 flow cell. Library preparation was performed without a size-selection step. Basecalling and read processing were conducted with Dorado (super-accurate mode; v7.9.8) (11) in EPI2ME (v5.2.5) (12); quality reports were generated using the EPI2ME workflow and NanoPlot (v1.46.2) (13). All software was run using the default parameters. Sequencing and assembly metrics are provided in Table 1.

**TABLE 1:** Genome assembly metrics, annotation features, and taxonomic assignment of four phyllosphere bacterial strains isolated from *Mangifera indica*

Strain	No. of contigs	Genome length (bp)^*a*^	GC content (%)	Coverage	Assembly N50	Read length N50	Total reads	Total bases sequenced	Predicted genes (PGAP)	Taxonomic assignment (ANI%, dDDH%)
T01M1	1	3,268,579	70.67	89×	3,268,579	6,859	77,913	303,085,341	3,054	*Microbacterium radiodurans* DSM 25,564 (97.1, 74.4)
T01M4	1	4,120,405	72.78	56×	4,120,405	8,215	53,800	240,131,496	3,883	*Brachybacterium rhamnosum* JCM 11,650 (96.0, 65.8)
RM6	2	4,353,817^*a*^	70.02	49×	4,198,739	7,936	56,431	231,256,580	3,993	*Sphingomonas citri* RRHST34 (98.5, 87.3)
RM9	2	3,535,914^*a*^	71.80	70×	3,474,087	8,732	54,600	262,676,049	3,401	*Curtobacterium caseinilyticum* GDMCC 1.2667T (87.2, 28.9); assigned as *Curtobacterium* sp.

^*a*^ RM6 comprises a 4,198,739-bp chromosome and a 155,078-bp plasmid, whereas RM9 comprises a 3,474,087-bp chromosome and a 61,827-bp plasmid.

Assemblies were generated and polished using Flye v2.9.5 (14), and two plasmid-associated contigs were identified in only two of the four strains. Genomes were circularized with Circlator (v1.5.5) (15) using dnaA as the starting gene. Species-level assignment used average nucleotide identity (ANI) with FastANI (v1.34) (16) and digital DNA-DNA hybridization (dDDH) values were calculated using the Genome-to-Genome Distance Calculator (GGDC) v3.0 (17). Genome annotation was performed using the NCBI Prokaryotic Genome Annotation Pipeline (PGAP) v6.10 (18) during genome submission to GenBank (Table 1).

Species-level analyses identified strains T01M1 and T01M4 as *Microbacterium radiodurans* and *Brachybacterium rhamnosum*, respectively, whereas strains RM6 and RM9 were assigned to *Sphingomonas citri* and *Curtobacterium* sp. Genomes ranged from 3.27 to 4.20 Mb, with GC contents of 70.02%–72.78% and sequencing coverage of 49×–89×.

## Data Availability

The genome assemblies and raw sequencing reads are publicly available in NCBI and Genbank. *M. radiodurans* is available under SRA accession number SRR37024946 and genome accession number GCA_054956835.1. This Whole Genome Shotgun project has been deposited at GenBank under the BioProject accession number PRJNA1415819, BioSample accession number SAMN54949484, and WGS master accession number JBUDZJ000000000. *B. rhamnosum* is available under SRA accession number SRR37025155 and genome accession number GCA_054957695.1. This Whole Genome Shotgun project has been deposited at GenBank under the BioProject accession number PRJNA1415832, BioSample accession number SAMN54949961, and WGS master accession number JBUDZI000000000. *S. citri* is available under SRA accession number SRR37025327 and genome accession number GCA_054917915.1. This Whole Genome Shotgun project has been deposited at GenBank under the BioProject accession number PRJNA1415841, BioSample accession number SAMN54950128, and WGS master accession number JBUBWT000000000. The sequences for the chromosome and plasmid of *S. citri* sp. RM6 were deposited in GenBank under accession numbers JBUBWT010000001 and JBUBWT010000002, respectively. *Curtobacterium* sp. is available under SRA accession number SRR37038865 and genome accession number GCA_054956955.1. This Whole Genome Shotgun project has been deposited at GenBank under the BioProject accession number PRJNA1416258, BioSample accession number SAMN54958354, and WGS master accession number JBUDZK000000000. The sequences for the chromosome and plasmid of *Curtobacterium* sp. RM9 were deposited in GenBank under accession numbers JBUDZK010000001 and JBUDZK010000002, respectively. In addition, plasmid-associated contigs corresponding to the second assembly contigs of *S. citri* RM6 and *Curtobacterium* sp. RM9 are available under accession numbers NZ_JBUBWT010000002 and NZ_JBUDZK010000002, respectively.
